# Horizontal and Vertical Distance Perception in Altered Gravity

**DOI:** 10.1038/s41598-020-62405-0

**Published:** 2020-03-25

**Authors:** Gilles Clément, Angie Bukley, Nuno Loureiro, Louise Lindblad, Duarte Sousa, André Zandvilet

**Affiliations:** 1 Lyon Neuroscience Research Center, Bron, France; 2 International Space University Org., Inc., Webster, Massachusetts, USA; 3 Champalimaud Research, Champalimaud Centre for the Unknown, Lisbon, Portugal; 4 Galactic Purpose Unipessoal Lda, Lisbon, Portugal; 5 OHB, Bremen, Germany; 6 European Space Research and Technology Center, Noordwijk, The Netherlands

**Keywords:** Neuroscience, Cognitive neuroscience, Perception

## Abstract

The perception of the horizontal and vertical distances of a visual target to an observer was investigated in parabolic flight during alternating short periods of normal gravity (1 g). microgravity (0 g), and hypergravity (1.8 g). The methods used for obtaining absolute judgments of egocentric distance included verbal reports and visually directed motion toward a memorized visual target by pulling on a rope with the arms (blind pulling). The results showed that, for all gravity levels, the verbal reports of distance judgments were accurate for targets located between 0.6 and 6.0 m. During blind pulling, subjects underestimated horizontal distances as distances increased, and this underestimation decreased in 0 g. Vertical distances for up targets were overestimated and vertical distances for down targets were underestimated in both 1 g and 1.8 g. This vertical asymmetry was absent in 0 g. The results of the present study confirm that blind pulling and verbal reports are independently influenced by gravity. The changes in distance judgments during blind pulling in 0 g compared to 1 g support the view that, during an action-based task, subjects base their perception of distance on the estimated motor effort of navigating to the perceived object.

## Introduction

The accurate perception of visual space is fundamental for orientation and locomotion. Grüsser^1^ defined two major regions of perceptual space: personal space and extra-personal space. Extra-personal space is further divided into four action spaces: distances up to 2 m comprise grasping space; distances up to 8 m comprise the near-distant action space; distances up to 30 m comprise the far-distant action space; and finally, vista space is everything in the visual background that is beyond 30 m. Experimental results indicate that egocentric distances are accurately judged in the near-distant action space^2^. In this space, depth cues, such as convergence, accommodation, as well as binocular stereo can provide accurate absolute distance information that is required for reaching, for instance. In the near-distant action space, locomotion control is aided by the eye-height scaled perspective, which provides accurately scaled egocentric distance^3^.

Perceived accurate absolute distance in the near-distant action space is particularly important for fall prevention. Several studies have shown that the perceived egocentric distance in the near-distant action space was influenced by the anticipated effort required to perform the task and by the physiological status of the observers^4–9^. For example, regardless of the actual target distance, subjects wearing a heavy backpack perceive greater distances than other subjects without a backpack^4^, people with large body sizes perceive greater distance than other people^5,6^. Also, older subjects perceive greater egocentric distance than younger subjects, and this difference is not due to age-related difference in the use of eye height or texture gradient information^7,8^. An explanation for these differences is that people with heavy backpack, bigger sizes, and the elderly, anticipate greater effort (as if imagining that they were walking to the target) than other people and thus bias their distance judgment consistent with expected effort^9^.

The perception of vertical distance (height) has received much less attention than the perception of horizontal distance^10^. However, in agreement with the effort-based explanation above, the *gravity theory* predicts that, in the action space, vertical distances will be judged as longer when viewed from the bottom of a vertical surface than they are from the top. This is because ascending requires more energy expenditure than descending. When one ascends, muscle contractions are required to pull the body weight up to a specific point. However, descending involves relaxing muscles for a partially controlled series of drops in the direction of gravity^11^. Consequently, the gravity theory predicts shorter distance judgments when looking down than when looking up because it requires less energy to descend than to ascend.

We tested the prediction from the gravity theory in experiments in which we compared the judgments of egocentric distance within the near-distant space in normal gravity (1 g), in microgravity (0 g), and in hypergravity (1.8 g). Compared to 1 g, shorter distance judgments were expected in 0 g because moving in 0 g requires less muscular effort, and longer distances judgments were expected in 1.8 g because moving in 1.8 g requires more muscular effort. We also compared the judgments of egocentric distance when looking up and down in various gravity levels. The hypothesis was that distance judgments when looking down are shorter than when looking up in 1 g and 1.8 g (because less effort is required when going down than going up), but that this asymmetry is absent in 0 g (because the same muscular effort is required when going up and down).

Non-action based tasks and action-based tasks can be used to measure egocentric distance perception. Non-action based tasks typically include a verbal report with explicit distance scales, such as meters of feet. Action-based tasks usually include blind walking, blind rope pulling, or blind throwing to a previously seen target^7,12,13^. Prior research activities have revealed that different results are obtained when the two types of tasks were executed. We therefore used both task types, the verbal report of distance and blind pulling, to assess the effect of gravity on egocentric distance perception. The fact that it is forbidden for test subjects to walk during the 1.8 g pull-ups and pull-outs of parabolic flights for safety reasons coupled with the impossibility for subjects to walk during in 0 g (their feet don’t contact the floor in free-fall) drove our decision to use blind pulling. Previous experiments have shown that the responses from blind pulling correlate strongly with those of blind walking^14,15^. Both blind pulling and blind walking are open-loop processes based on one initial visual input that the brain processes to estimate the distance to be traveled. Being blindfolded, the test subjects must rely on proprioceptive and vestibular cues in concert with efferent copy to update body movement. Based only on this sensorimotor information, subjects reproduce distances of up to 9 m fairly accurately in 1 g^14^. By using both verbal reports and blind pulling, we were able to examine whether the ability to judge egocentric distance in the horizontal and vertical directions was influenced by the gravity level and the expected muscular effort.

## Methods

### Participants

Nine subjects (7 males, 2 females) aged from 23 to 62 (mean 41.7 years) participated in these studies. Distance judgments were measured during two campaigns of parabolic flight (186 parabolas) on board the Novespace Airbus 300 Zero G aircraft. The experiment was undertaken with the informed and written consent of each subject for both study participation and publication of image in an online open-access publication. The test procedures were approved by the European Space Agency medical board (Cologne, Germany) and by the *Comité de Protection des Personnes Nord Ouest III* (Caen, France), and were performed in accordance with the ethical standards laid down in the 1964 Declaration of Helsinki.

### Equipment

**Figure 1:**
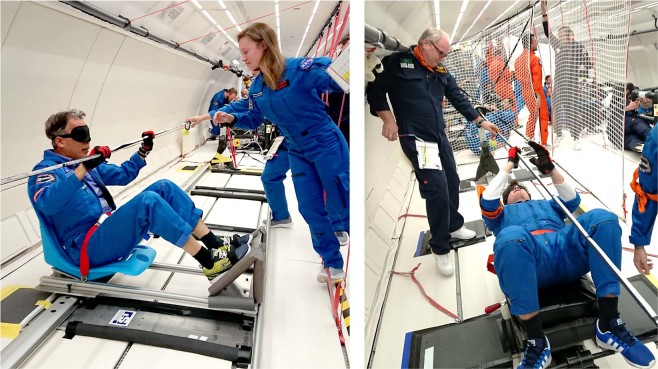
Experimental set-up for measuring lateral (left) and vertical (right) distance judgments. Subjects were either sitting upright and moving to their right/left or lying on their back and moving up/down by pulling on a rope. Photos courtesy of Novespace.

The equipment included a visual target (a tennis ball) that was positioned at different distances from the subject at eye level, and a sled mounted on a 7-m long rail attached to the aircraft floor. The rail was attached to the aircraft’s seat tracks, along the x-direction of the aircraft, using supporting beams and nut fittings. The subjects moved along the rail by pulling on a rope that was tensioned with turnbuckles to vertical beams attached at both ends of the rail above the subjects’ head^15^. For horizontal distance judgments, the subjects were seated upright and translated sideways to the right or left (Fig. 1, left). For vertical distance judgments, the subjects were lying on their back and translated upward or downward (Fig. 1, right).

### Experimental protocol

An experimental trial started with the subject wearing a blindfold with his right/left shoulder or head/feet against one of the vertical beams. An operator suspended a tennis ball on the rope at eye level at a predetermined distance. The subject lifted the blindfold, looked at the tennis ball, returned the blindfold, and verbally reported the perceived distance (in m and cm). The subject then pulled the rope to move the seat until she thought her nose was the closest to the tennis ball (in the meantime, the operator had removed the tennis ball). One operator noted the actual position of the visual target, the verbal estimate, and the final position of the subject. Another operator noted the time for the subject to complete the trial. The subject then returned with the sled to the original position by hand-pulls. At no point during the test did the subjects receive a feedback on their distance estimate performance. Two video cameras recorded the experiment in its entirety^15^.

Each subject was tested during 20 parabolas in 1 g, 0 g, and 1.8 g. Subjects were asked to judge 10 distances (ranging from 0.6 m to 6.0 m) in the lateral directions (right and left) and 10 distances (ranging from 0.6 m to 5.0 m) in the vertical directions (up and down). Because the operators were also used as subjects the target distances were different across subjects. The subjects did not receive any feedback on their performance. The order of the target distances was also randomized across gravity levels and subjects.

Each parabola started with the aircraft flying in a straight and level flight at 1 g for 1 minute, followed by a pull up phase at 1.8 g, then a free-fall phase (0 g), and ended with a pull out phase at 1.8 g. The 1.8 g and 0 g phases lasted about 23 sec. During each parabola, subjects were tested during one trial in the 1 g phase, one trial in the 0 g phase, and one trial in the 1.8 g pull out phase. All subjects had previous experience of parabolic flight. Six subjects took prophylactic medication (a combination of promethazine and dextroamphetamine) before boarding the plane, and none of them showed symptoms of motion sickness during the flight. These medications are well known for their drowsiness effects, which could affect the subjects’ performance. However, *c*ontrol measurements in 1 g were performed on board the aircraft during straight and level flight between successive parabolas, while the medicated subjects were under the influence of the drug. This was to ensure that the changes seen across the various gravity levels were not due to the effect of the medication^15^.

### Data analysis

The distance judgments were plotted as function of true target distances for each subject, gravity level, and direction. For each condition, the errors between actual target distances and distance judgments were calculated. The percent asymmetry in distance judgments was calculated as the difference in the distance judgment error between left/right targets and between up/down targets.

Because previous studies have shown that the speed of blind walking increased as participants gained confidence with the repetition of trials^16^, we also measured the duration of each trial and calculated the speed of motion during blind pulling.

The distance traveled or reported was fitted by the output of Lappe *et al*.^17^ leaky spatial integrator model. For the task of moving to a previously seen distance, this model predicts perceived distance (x) at which the subject believed they had reached the target for a given target distance (d) according to the following equation:

1 $${\rm{x}}=-\,(1/{\rm{a}})\,\ast \,\mathrm{ln}({\rm{k}}/({\rm{d}}\,\ast \,{\rm{a}}+{\rm{k}}))$$

where *k* is the sensory gain (k = 1 for an ideal observer) and *a* represents the leaky integrator constant or leak rate (a = 0 for an ideal observer)^18^.

The mean distance error, percent asymmetry, and speed of motion were then binned for 5 classes of distances for the horizontal distance tests (0.6–1.6 m; 1.7–2.7 m; 2.8–3.8 m; 3.9–4.9 m; 5.0–6.0 m) and the vertical distance tests (0.6–1.4 m; 1.5–2.3 m; 2.4–3.2 m; 3.3–4.1 m; 4.2–5.0 m).

The binned distance error, percent asymmetry, and speed of motion were compared across the 3 gravity levels with repeated measures ANOVAs in Excel. Using an alpha error of 0.05 as the decision rule, the null hypothesis was that there is no difference across gravity level and target distance. Tests of distribution and homogeneity of variances were also performed to check the normality assumption for ANOVAs.

## Results

### Horizontal (lateral) distance perception

**Figure 2:**
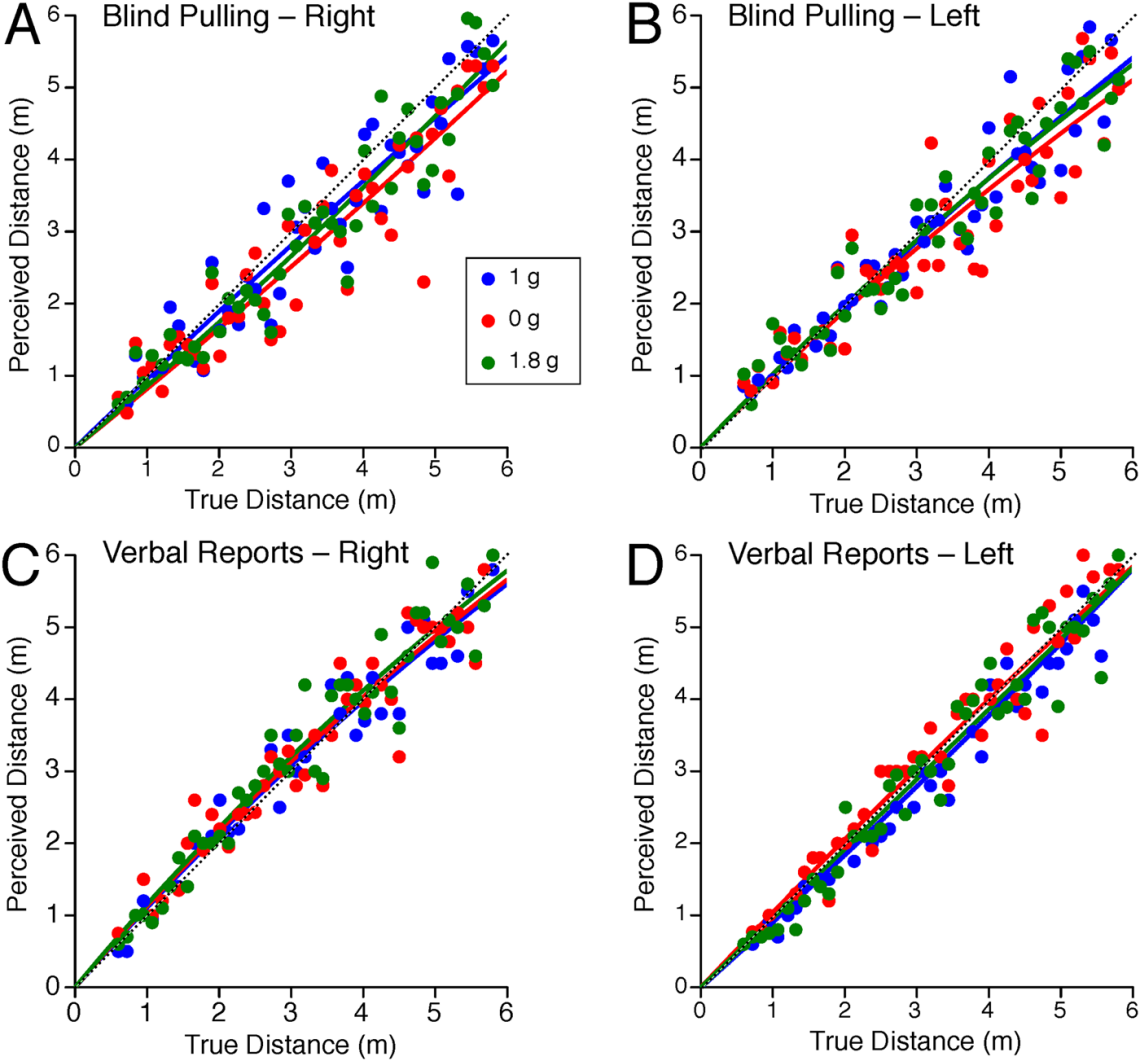
Lateral distance judgments. Each dot represents an individual distance estimate during blind pulling to right (**A**) and left (**B**) targets, and during verbal reports to right (**C**) and left (**D**) targets in the 3 gravity levels. The dotted line represents a perfect response. The distance traveled or reported was fitted by the output of Lappe *et al*.^17^ leaky spatial integrator model according to the equation [1]. The sensory gain (k) and leaky integrator constant (a) are reported in Table 1.

The distance judgments obtained for blind pulling and verbal reports during the horizontal distance tests are shown in Fig. 2. For all gravity levels, subjects accurately judged distances when using verbal reports, but generally underestimated distances when using blind pulling.

**Table 1:** Outputs (a and k) of Lappe *et al*.^17^ leaky spatial integrator model fit to the whole data set according to the equation [1] for vertical and lateral distances travelled and reported.

Methods	Direction	g level	a	k	R^2^
Blind pulling	right	1 g	0.024	1.030	0.872
		0 g	−0.029	1.238	0.856
		1.8 g	−0.039	1.188	0.904
Blind pulling	left	1 g	0.041	0.989	0.912
		0 g	0.065	0.988	0.841
		1.8 g	0.062	0.949	0.906
Verbal reporting	right	1 g	0.072	0.867	0.944
		0 g	0.077	0.847	0.929
		1.8 g	0.075	0.828	0.937
Verbal reporting	left	1 g	−0.027	1.116	0.956
		0 g	0.027	0.947	0.932
		1.8 g	−0.005	1.045	0.946
Blind pulling	up	1 g	0.144	0.705	0.894
		0 g	0.127	0.834	0.802
		1.8 g	0.099	0.841	0.824
Blind pulling	down	1 g	0.192	0.950	0.827
		0 g	0.145	0.861	0.863
		1.8 g	0.215	0.886	0.759
Verbal reporting	up	1 g	0.028	0.847	0.948
		0 g	0.102	0.718	0.949
		1.8 g	0.044	0.823	0.935
Verbal reporting	down	1 g	−0.013	1.097	0.947
		0 g	−0.029	1.119	0.910
		1.8 g	−0.033	1.151	0.930

R^2^ indicates how close the data are to the fitted regression line.

**Figure 3:**
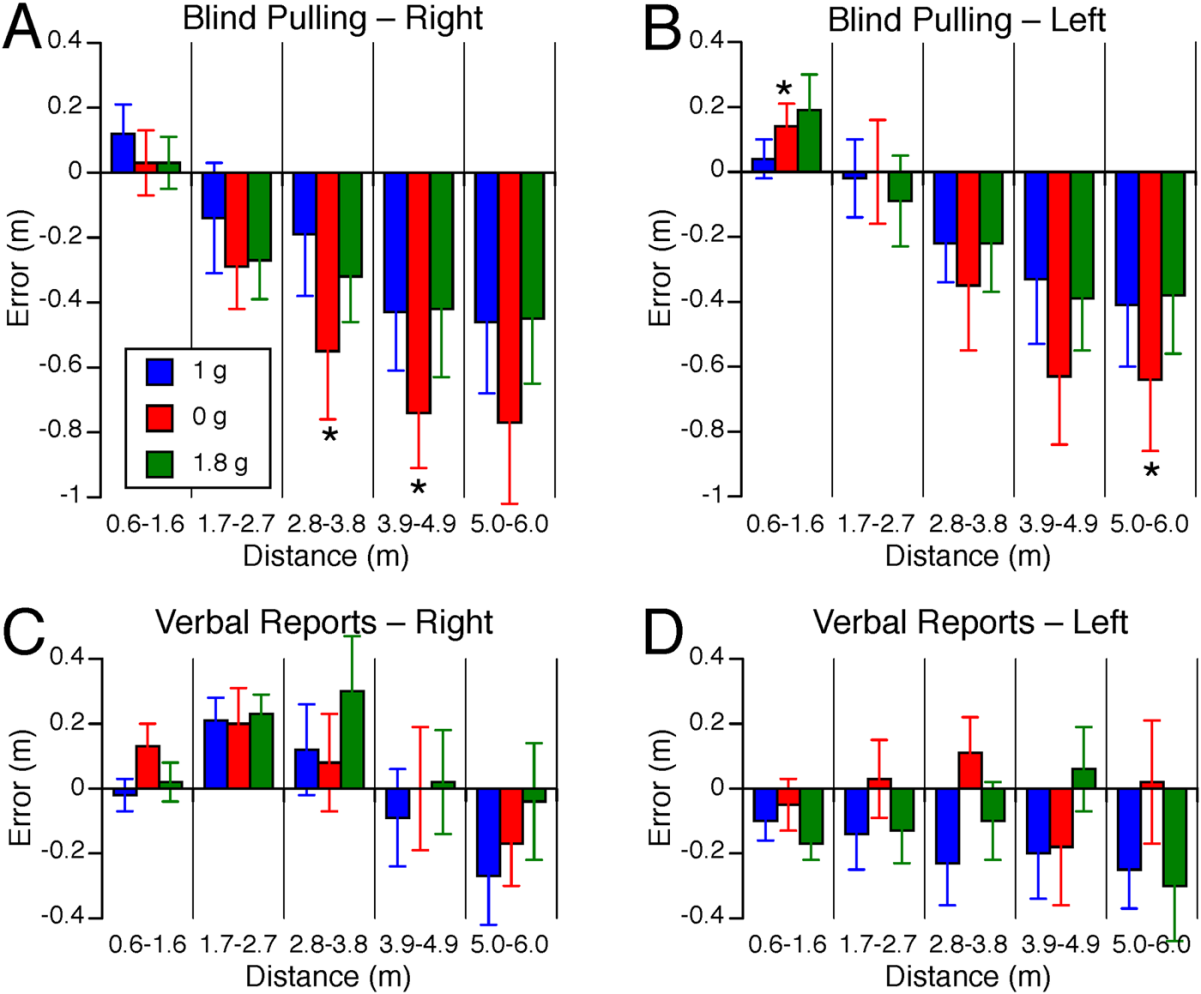
Error in lateral distance judgments. Mean ± SE of errors for the 9 subjects binned in 5 distance ranges during blind pulling to right (**A**) and left (**B**) targets, and during verbal reports to right (**C**) and left (**D**) targets in the 3 gravity levels. Positive error means that the subjects overestimated distance; negative error means that they underestimated distances. *P < 0.05 relative to 1 g; ^†^P < 0.05 relative to 0 g.

A repeated measures ANOVA with 2 factors (distance error: 5 bins; gravity level: 1 g, 0 g, 1.8 g) indicated that the error in distance judgments during blind pulling significantly changed with target distance for both the right targets [F(4,134) = 6.41, P < 0.001, Fig. 3A] and the left targets [F(4,134) = 8.02, P < 0.001, Fig. 3B]. The error in distance judgments during blind pulling was not significantly different across the 3 gravity levels for either the right [F(2,134) = 2.66, P = 0.07] or the left targets [F(2,134) = 0.84, P = 0.43]. However, a repeated measured ANOVA with 2 factors (distance error; 5 bins: gravity level: 1 g, 0 g) indicated that the error in distance judgments during blind pulling was significantly different in 0 g compared to 1 g for both the right targets [F(1,89) = 4.62, P = 0.03] and the left targets [F(1,89) = 4.00, P = 0.048]. Post hoc paired samples t-test corrected for multiple comparisons (Bonferroni) indicated that the error in distance judgments during blind pulling was significantly (P < 0.05) larger in 0 g compared to 1 g for the right targets at distances ranging from 2.8–4.9 m and for the left targets at distances ranging from 0.6–1.6 m and 5.0–6.0 m (Fig. 3A,B).

A repeated measures ANOVA with 2 factors (distance error: 5 bins; gravity level: 1 g, 0 g, 1.8 g) indicated that the error in distance judgments in verbal reports significantly changed with target distances when targets were presented to the subject’s right [F(4,134) = 4.01, P = 0.004] (Fig. 3C), but not to the subject’s left [F(4,134) = 0.32, P = 0.86] (Fig. 3D). There was no significant effect of gravity on the error in distance judgments in verbal reports for right targets [F(2,134) = 0.96, P = 0.39] and for left targets [F(2,134) = 2.38, P = 0.09].

**Figure 4:**
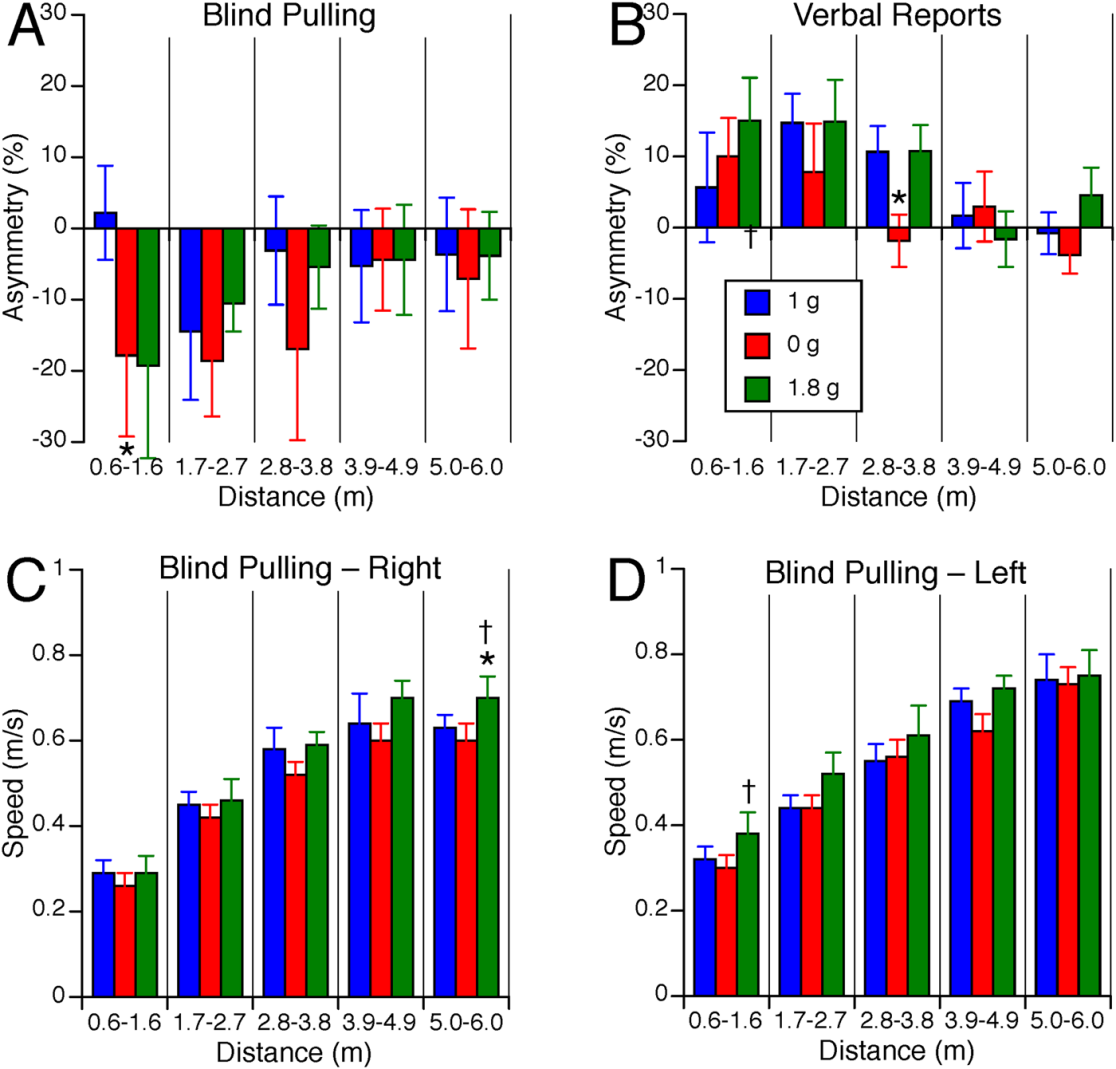
Asymmetry of horizontal distance judgments during blind pulling (**A**) and verbal reports (**B**). Positive asymmetry means that left targets were perceived farther than right targets. Speed of motion during blind pulling towards right (**C**) and left targets (**D**). Mean ± SE for the 9 subjects binned in 5 distance ranges during blind pulling. *P < 0.05 relative to 1 g; ^†^P < 0.05 relative to 0 g.

In 1 g, the average asymmetry between the distance judgments of the right and left targets during blind pulling ranged from 0% to 15% (Fig. 4A,B). During blind pulling, this right-left asymmetry did not change significantly with target distances [F(4,134) = 0.73, P = 0.58] or across the gravity levels [F(2,134) = 1.09, P = 0.34] (Fig. 4A). The right-left asymmetry in the error in distance judgments in verbal reports significantly decreased with target distances [F(4,134) = 3.87, P < 0.001], i.e. it went from a positive asymmetry for near distances (left targets were perceived farther than right targets) to no asymmetry for farther distances. However, this asymmetry was not affected by the gravity levels [F(2,134) = 1.73, P = 0.81] (Fig. 4B).

The duration of the trials ranged from 2.3 to 12.6 s. The speed of motion during blind pulling increased with target distance [right targets: F(4,134) = 43.58, P < 0.001; left targets: F(2,134) = 42.93, P < 0.001]. Motion speed was marginally significantly different across gravity levels [right targets: F(2,134) = 3.00, P = 0.053; left targets: F(2,134) = 3.04, P = 0.052] (Fig. 4C,D). Post hoc paired samples t-test corrected for multiple comparisons (Bonferroni) indicated that the speed of motion for the right targets increased significantly (P < 0.05) in 1.8 g compared to 1 g and 0 g for the longest distances (5.0–6.0 m). The speed of motion for the left targets increased significantly (P < 0.05) in 1.8 g compared to 0 g for the shortest distances (0.6–1.6 m).

### Vertical distance perception

**Figure 5:**
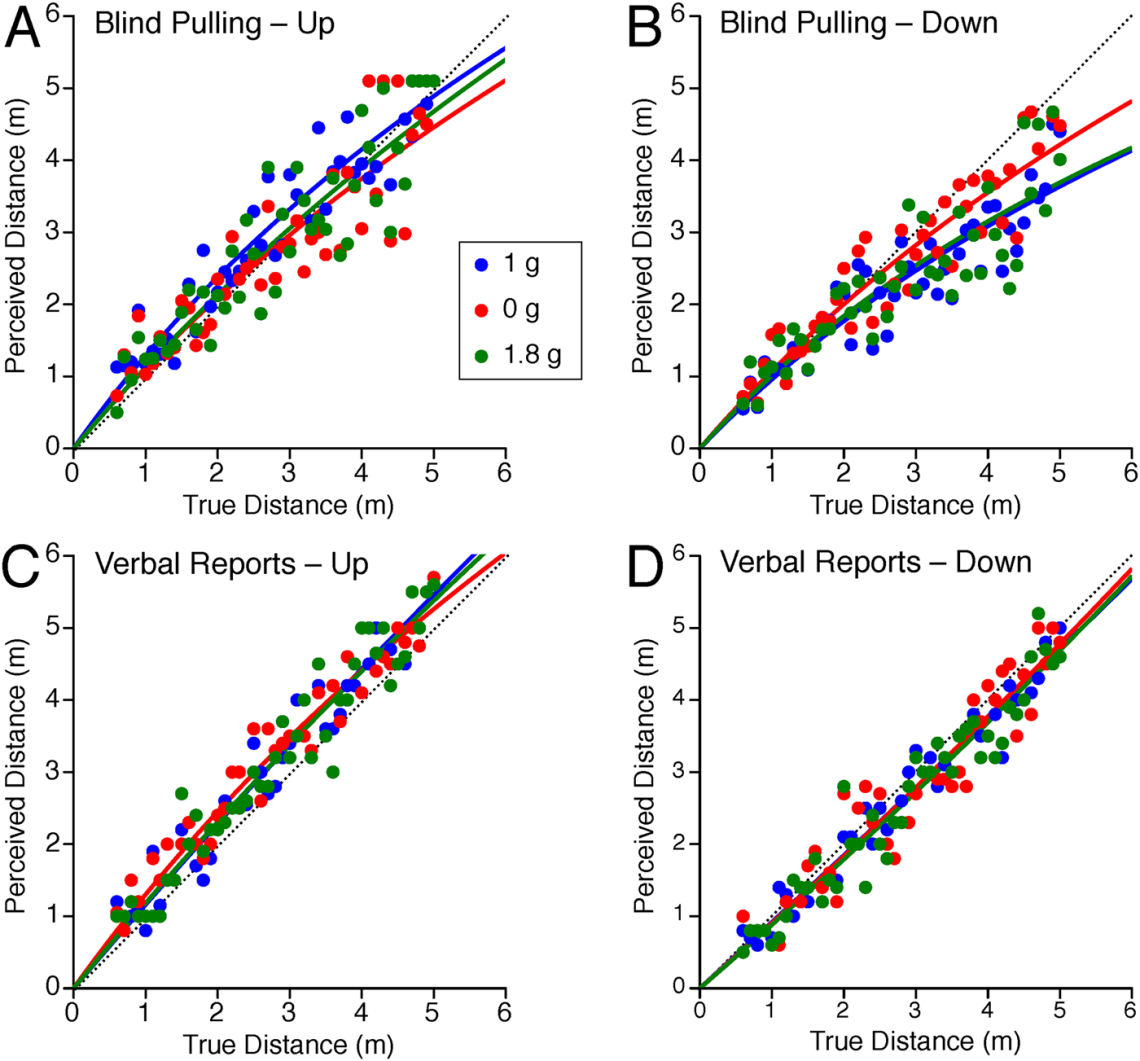
Vertical distance judgments. Each dot represents an individual distance estimate during blind pulling to up (**A**) and down (**B**) targets, and during verbal reports to up (**C**) and down (**D**) targets in the 3 gravity levels. The dotted line represents a perfect response. The distance traveled or reported was fitted by the output of Lappe *et al*.^17^ leaky spatial integrator model according to the equation [1]. The sensory gain (k) and leaky integrator constant (**a**) are reported in Table 1.

The distance judgments obtained for blind pulling and verbal reports during the vertical distance tests are shown in Fig. 5. For all gravity levels, subjects overestimated upward distances and underestimated downward distances in their verbal reports. During blind pulling, subjects also overestimated upward distances and underestimated downward distances in 1 g.

**Figure 6:**
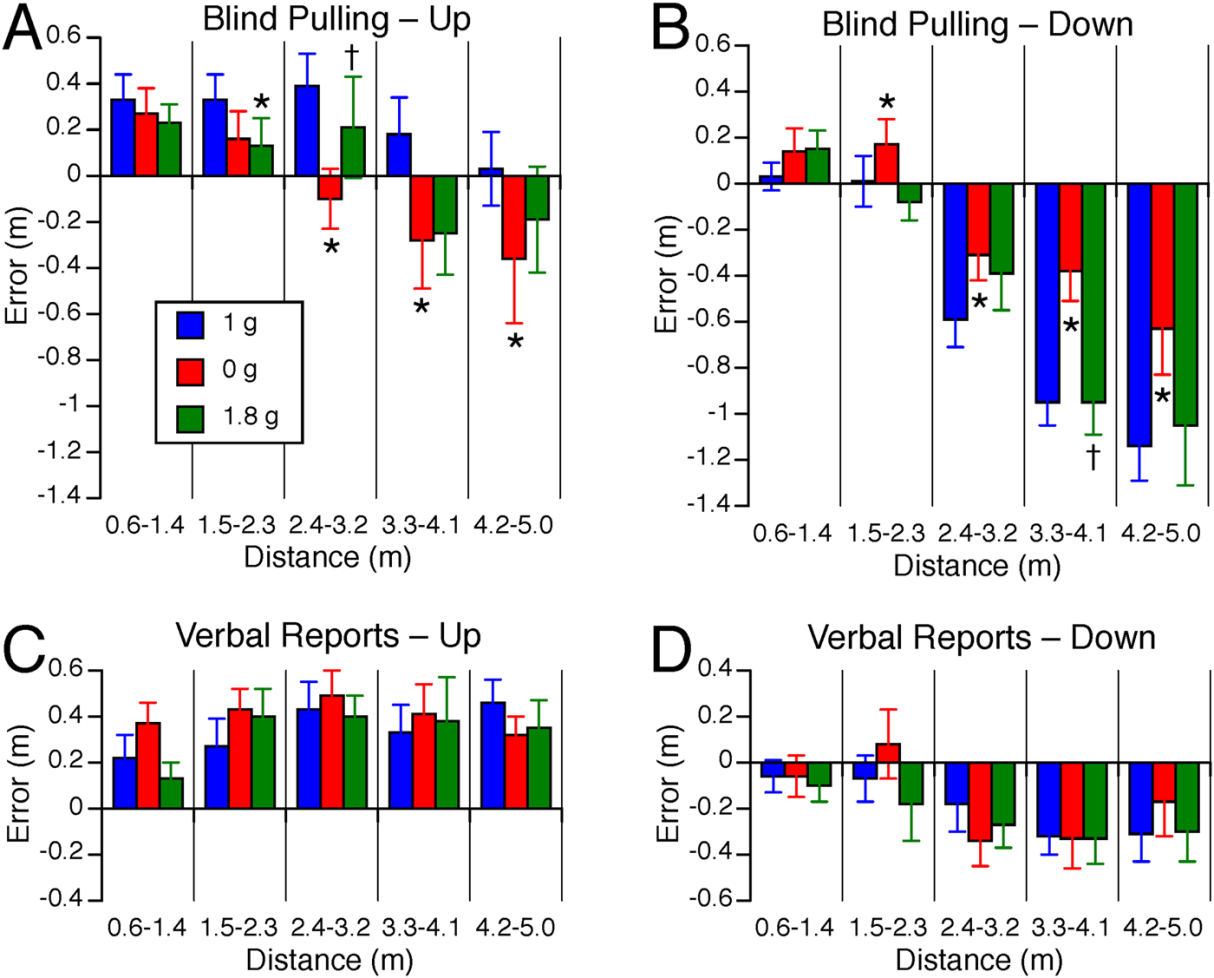
Error in vertical distance judgments. Mean ± SE of errors for the 9 subjects binned in 5 distance ranges during blind pulling to up (**A**) and down (**B**) targets, and during verbal reports to up (**C**) and down (**D**) targets in the 3 gravity levels. Positive error means that the subjects overestimated distance; negative error means that they underestimated distances. *P < 0.05 relative to 1 g; ^†^P < 0.05 relative to 0 g.

When targets were presented to the subject’s up or down, a repeated measures ANOVA with 2 factors (distance: 5 bins; gravity level: 1 g, 0 g, 1.8 g) indicated that the error in distance judgments during blind pulling significantly changed with target distance [up targets: F(4,134) = 4.46, P < 0.01; down targets: F(4,134) = 35.10, P < 0.001] (Fig. 6A,B). There was also a statistically significant difference in the error in distance judgments during blind pulling between the gravity levels [up targets: F(2,134) = 4.69, P = 0.01; down targets: F(2,134) = 8.02, P < 0.001]. Distances of up targets ranging from 2.4–3.2 m, 3.3–4.1 m, and 4.2–5.0 m were underestimated in 0 g compared to 1 g (P < 0.05). Distances of up targets ranging from 2.4–3.2 m were also underestimated in 0 g compared to 1.8 g (P < 0.05). By contrast, distances of down targets ranging from 2.4–5.0 m were less underestimated (the error in distance judgment decreased) in 0 g compared to 1 g (P < 0.05).

The error in distance judgments in verbal reports changed significantly with target distances when targets were presented to the subject’s down direction [F(4,134) = 3.53, P < 0.01] (Fig. 6D), but not to the subject’s up direction [F(4,134) = 1.12, P = 0.31] (Fig. 6C). There was no significant effect of gravity on the error in distance judgments in verbal reports for up targets [F(2,134) = 0.61, P = 0.54] and for down direction targets [F(2,134) = 0.49, P = 0.62].

**Figure 7:**
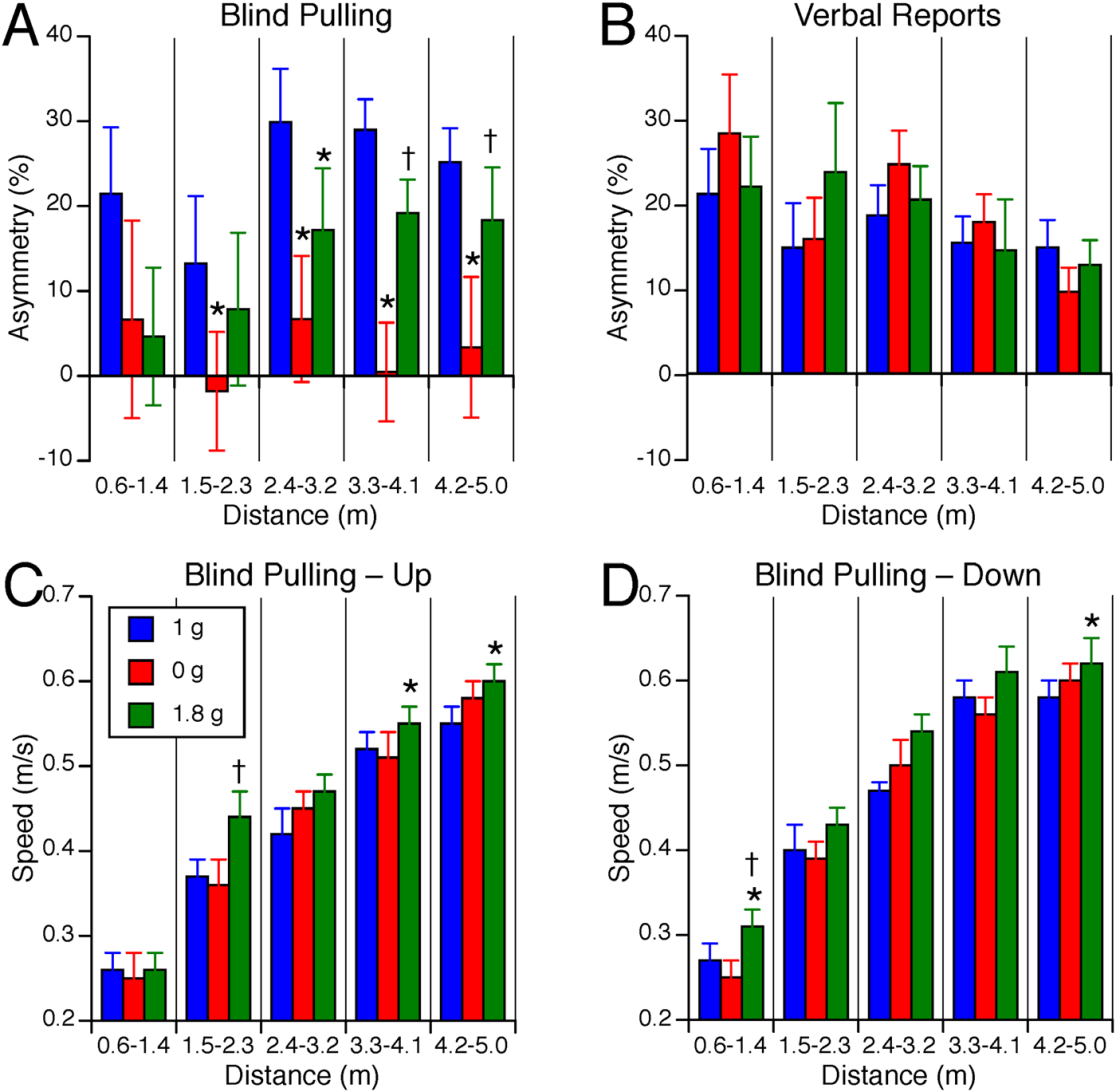
Asymmetry of vertical distance judgments during blind pulling (**A**) and verbal reports (**B**). Positive asymmetry means that up targets were perceived farther than down targets. Speed of motion during blind pulling towards up (**C**) and down targets (**D**). Mean ± SE for the 9 subjects binned in 5 distance ranges during blind pulling. *P < 0.05 relative to 1 g; ^†^P < 0.05 relative to 0 g.

In 1 g, the average asymmetry in distance judgments between the up and the down targets ranged from 15% to 30%, depending on target distance (Fig. 7A,B). During blind pulling, the up-down asymmetry in the error in distance judgments was significantly different across the gravity levels [F(2,134) = 10.17, P < 0.001]. This vertical asymmetry did not significantly changed with target distances [F(4,134) = 1.26, P = 0.29]. Post hoc paired samples t-test corrected for multiple comparisons (Bonferroni) indicated that the up-down asymmetry significantly decreased in 0 g compared to 1 g for all target distances except for the shortest distances (Fig. 7A). The up-down asymmetry also significantly decreased in 0 g compared to 1.8 g for the longest distances (3.3–4.1 m and 4.2–5.0 m). During verbal reports, the up-down asymmetry in the error in distance judgments significantly changed with target distances [F(4,134) = 2.50, P = 0.05], but stayed the same across all three gravity levels [F(2,134) = 0.29, P = 0.74] (Fig. 7B).

The duration of the trials ranged from 2.0 to 10.1 s. The speed of motion during blind pulling significantly increased with target distance [up targets F(4,134) = 82.71, P < 0.001; down targets: F(4,134) = 99.9, P < 0.001] and was significantly different across gravity levels [up targets: F(2,134) = 4.12, P = 0.02; down targets: F(2,134) = 5.60, P < 0.01]. Post hoc paired samples t-test corrected for multiple comparisons (Bonferroni) indicated that the speed of blind pulling significantly increased (P < 0.05) in 1.8 g compared to 1 g for the up targets ranging from 3.3–4.1 m and 4.2–5.0 m, and in 1.8 g compared to 0 g for the up targets ranging from 1.5–2.3 m (Fig. 7C). The speed also significantly increased in 1.8 g compared to 1 g for the down targets at both the shortest and longest distances, and in 1.8 g compared to 0 g for the shortest distances (Fig. 7D).

## Discussion

The results of this study indicate that there is an asymmetry in vertical distance judgments in the action space in normal gravity: distances of up targets are overestimated, whereas distances of down targets are underestimated. This asymmetry was present in both verbal reports and blind pulling judgments in both 1 g and 1.8 g. In 0 g, the up-down asymmetry of distance judgments was present in verbal reports, but not during blind pulling.

Horizontal distance judgments were symmetrical in 1 g, and changes in the gravity level did not alter this symmetry during either blind pulling or verbal reporting. However, overall horizontal distance judgments were underestimated in 0 g compared to 1 g. This result is in agreement with our previous study where sitting subjects judged the distance of targets presented straight-ahead and were blind pulling in the forward direction^15^. In this previous study, distances were also underestimated in 0 g compared to 1 g during blind pulling, and to a lesser degree in verbal reports. The results from the present study confirm that horizontal distance judgments during blind pulling are more affected by 0 g than those using verbal reports.

### Difference between verbal reports and blind pulling

The cognitive mechanisms used for pulled distances and verbal reports are quite different. Both mechanisms require an estimation of the distance between the target object and the observer. The verbal report requires converting a perceived visual distance in an abstract reference frame (meters or feet), which is influenced by the environmental setting coupled with the experience and expectations of the subject^19^. On the other hand, pulled distance requires converting the perceived distance into a physical action (moving the body), which involves the otoliths, proprioceptive stimulation, and motor commands^15^. Of the two responses, blind pulling is the more natural response because no mental transformation of the target location is required. Subjects use their own internal reference frame to determine the location of the target^20^. As a result, verbal reports are generally less accurate than blind-walking judgments: previous studies have shown that verbal reports are typically underestimated for distances greater than 3 m^12,13^, as is the case in the present study.

Some authors have argued that a single underlying representation is responsible for perception, whether a participant is asked to generate a verbal estimate or walk blindfolded to the previously seen target^14^. Alternatively, other researchers argue for two distinct internal representations of perceived distance: one allocentric related to object location in spatial references, and one egocentric linked to individual action-based tasks^21^. The results of the present study and our previous study^15^ showing that blind pulling and verbal reports are independently influenced by gravity support the view that different representations underlie the performance of the two tasks.

### Asymmetry of vertical distance perception

Two theories have been put forward to explain the asymmetry in vertical distance perception in the action space. The assumption that the estimated motor effort required to reach a perceived object or target influences distance perception is the basis for the *gravity theory*^4^. Consistent with this assumption, upward distances are over estimated and downward distances are underestimated. Another theory known as the *evolved navigation theory* is based on the idea that there is an evolutionary advantage in overestimating the risk of falling^22^. During descent, posture is less stable and visibility is reduced. As a result, falls occur more often during a descent than during ascending. Contrary to the gravity theory, this theory says that downward distances are overestimated. In 1 g, we found that upward distances were overestimated and downward distances were underestimated. The observed effect is in the direction predicted by the gravity theory, and opposite to the prediction of the evolved navigation theory. In 0 g, the same effort is generated for moving up as for moving down. Therefore, the lack of asymmetry in the perception of vertical distances seen in 0 g is also in agreement with the gravity theory.

Several studies have shown that energetic costs are associated with the asymmetry of vertical distance judgments. For example, for individuals carrying a heavy backpack, distances appear to be farther and hills appear to be steeper^4^. To people who are heavy, staircases appear to be steeper than they do to lighter individuals^8^. The same is true for individuals who are fatigued^23^. Objects on a ground appear farther to observers who intend to throw a heavy ball compared with observers who throw a light ball^9^. An up-hill walk of a given distance requires more energy, hence appears to be farther away as compared to the same distance to be covered on flat ground^24^.

Interestingly, at each gravity level that we tested, the motor effort deployed by the subject was the same when moving “up” or “down”, because they were moving along a direction that was perpendicular to gravity. The observed up-down asymmetry in their verbal reports and blind pulling in 1 g suggests that it is the estimated motor effort required for moving along a *mental representation* of the vertical direction aligned with gravity that is asymmetrical. In absence of this gravitational reference in 0 g, the spatial orientation system switches from an allocentric reference to an egocentric ref. ^25^. The brain then determines that the effort for moving up is the same as for moving down, and the blind pulling judgments become symmetrical.

The “paternalistic vision hypothesis” of the gravity theory, which proposes that visual perception distorts the world according to the perceiver’s abilities, has been strongly criticized recently^26^. An alternate interpretation for our finding is the possible differences in sensitivity to horizontal and vertical motions, and to the asymmetric sensitivity to upward and downward vertical motion^27,28^. The pioneering work of Fernandez & Goldberg^29^ demonstrated that the utricular sensitivity to horizontal plane motion in monkeys was higher than the saccular sensitivity to vertical plane motion. However, this result has been challenged by recent studies^30,31^ which showed that the monkey otolith afferents had similar gains during active and passive horizontal and vertical translations. Psychophysical studies, on the other hand, have reported that perceptual sensitivity was higher for vertical as compared to horizontal translations, and higher for downward as compared to upward translations^27,28^. The discrepancy between neurophysiologic data and perception data could reside in higher level processing of the vestibular signals, in multisensory integration or in cognitive factors.

Previous space experiments have shown that the asymmetry of the velocity of vertical eye movements, arm movements, and self-motion perception commonly observed in 1 g was no longer present in 0 g^32–34^. This vertical asymmetry in 1 g is also present when subjects are supine, which suggests that this asymmetry is not only due to the slowing of movements when counteracting gravity during an upward movement. Asymmetries in the perception of upward and downward motion reflect high-level processes in the central neural system, where higher sensitivity to the perception of downward motion would help detecting falls and maintaining balance^27,28^. It has been proposed that this vertical asymmetry is also due to an internal model of gravity that maintains invariant motor trajectories for effort optimization in Earth’s gravity^35^.

### Effects of gravity on distance judgments during blind pulling

We did not observe an increase in perceived distance in 1.8 g compared to 1 g during both horizontal and vertical blind pulling. This result did not confirm the findings from Proffitt *et al*.^14^ that subjects carrying a heavy backpack on Earth overestimate distance for distances exceeding 4 m. This discrepancy could be due to a different strategy used by our subjects in 1.8 g. At all three gravity levels, our subjects were pulling faster as the distance of the targets increased, presumably because they felt pressed by the time constraints of parabolic flight. The speed of blind pulling was not different between 1 g and 0 g for both the lateral (Fig. 4C,D) and vertical (Fig. 7C,D) distances. Therefore, the change in the asymmetry of vertical distances in 0 g (Fig. 7A) was not due to a change in the speed of motion during blind pulling. However, the speed of blind pulling was faster in 1.8 g than in 1 g for distances exceeding 4 m (Figs. 4C and 7C,D). For these distances, our subjects completed the blind pulling task in 7.2 s in 1.8 g, compared to 7.7 s in 1 g. This difference in speed could explain why our subjects did not overestimate distances in 1.8 g for the longest distances tested. Interestingly, Thomson^12^ had shown that blind walking responses were very accurate when completed in less than 8 s, and declined rapidly in accuracy when taking longer than 8 s. He interpreted these results in terms of representations of space in memory that remain at high-fidelity for 8 s.

In agreement with previous studies^36–41^, our results confirm that, in addition to visual depth cues, the otoliths, proprioceptive, and haptic cues also contribute to distance perception. For instance, when subjects invert their heads upside-down, the distance of targets viewed from between the legs is perceived longer than distances observed when subjects stand upright^38,39^. Subjects with their body upright and their head tilted upward overestimate the distance of objects presented above the horizon; when their head is tilted downward, they underestimate the distance^41^. Similarly, when the body is tilted backward, the perceived length of a rod is overestimated meaning that the distance to the wall appeared to be shorter^40^. When the body is tilted forward, this causes errors in the reaching movement because subjects underestimate the distance between their body and the virtual object^42^.

The results from these ground-based studies indicate that gaze, head, and body positions relative to gravity contribute to perceived distance. In the normal upright posture, the eyes are in the primary position, and the head and trunk are kept upright with respect to the direction of the gravity. Raising or lowering the eyes produces unusual proprioceptive information of the eye. Also, tilting the head upward while keeping the trunk erect produces unusual proprioceptive information of the neck, as well as different static otolith stimulation. Similarly, bending the trunk produces unusual proprioceptive information of the trunk and otolith stimulation.

These findings have important implications for spaceflight. The direction of gaze and body posture are altered in microgravity during spaceflight and after return to Earth gravity^25^. Perceptual distortions of three-dimensional space have been observed in microgravity during parabolic^43^ and orbital flight^44^, as well as in patients with vestibular disorders^45^ and in healthy subjects during galvanic vestibular stimulation^41^. In space, astronauts become increasingly dependent on their vision for spatial orientation because the proprioceptive and static otolithic signals are altered in reduced gravity. Therefore, a risk to astronauts working on the surface of the Moon or Mars could be the misperception of distance^46^. Crewmembers should be made aware of potential underestimation of the distance of objects in reduced gravity and devices for measuring distance such as radar distance measurement, laser range, or GPS should be provided as countermeasures.

## Data Availability

All data generated or analyzed during this study are included in this article.
